# Birth weight reference for Japanese twins and risk factors for infant mortality: A population-based study

**DOI:** 10.1371/journal.pone.0271440

**Published:** 2022-07-14

**Authors:** Yuri Ishida, Yo Takemoto, Masaya Kato, Mahbub Latif, Erika Ota, Naho Morisaki, Atsuo Itakura

**Affiliations:** 1 Department of Obstetrics and Gynecology, Graduate School, Juntendo University, Bunkyo, Tokyo, Japan; 2 St. Luke’s International University, Graduate School of Nursing Science, Chuo, Tokyo, Japan; 3 University of Dhaka, Institute of Statistical Research and Training, Dhaka, Bangladesh; 4 Tokyo Foundation for Policy Research, Minato, Tokyo, Japan; 5 Department of Social Medicine, National Research Institute for Child Health and Development, Setagaya, Tokyo, Japan; Universite Clermont Auvergne, FRANCE

## Abstract

There is no standard birth weight curve for twins in Japan other than a prototype curve based on 1988–1991. Twins have a high perinatal mortality rate than singletons; therefore, we developed a new standard curve for twin birth weight using data from the 1995–2016 Vital Statistics and compared it with previous reports. We used 469,064 cases for analysis, excluding stillbirths and cases with missing values, and created a standard curve using LMS (statistical methods to vary the distribution by using skewness, median, and coefficient of variation) method. In comparison with previous reports, the mean birth weight decreased by 100–200 g. The groups with the lowest neonatal death rates (NDRs) and infant death rates (IDRs) were those with a birth weight of 1,500–2,499 g (NDR: 0.3%, IDR: 0.6%) and those born at 34–36 weeks (NDR: 0.2%, IDR: 0.4%). Compared to these, the IDR was significantly higher in the 2,500–3,999 g group and the 37–39 weeks group (incidence rate ratio (IRR): 1.1 in the 2,500–3,999 g group, IRR: 1.3 in the 37w0d–39w6d group). In particular, the risks of neonatal mortality and infant mortality were higher in infants born at a birth weight above 3,500 g. Infants born at a birth weight above 3,500 g may include recipients of twin-to-twin transfusion syndrome. The most common causes of infant mortality are accidental death and sudden infant death syndrome (SIDS). We considered the possibility that infants treated as healthy newborns and whose mothers were discharged from the hospital without adequate twin care guidance may be more likely to experience unintentional accidents and SIDS at home. The present study suggested that creating a new twin birth weight standard curve and guidance on managing twins at home for full-term and normal birth weight infants may lead to a reduction in infant deaths.

## Introduction

In Japan, the probability of multiple births, especially twins, has risen in recent years [1,2]. In the last 30 years, the probability of having twins has increased 1.6 times, a trend that has also been observed in Western countries [1,3]. One of the possible causes for this increase is the rise in the number of older mothers, which is related to an increase in fertility treatments, such as ovulation induction and assisted reproductive technologies (ART) [3]. In Japan, the probability of a spontaneous pregnancy resulting in dichorionic diamniotic twins (DD twins) is 0.2%–0.3%, and the probability of monochorionic diamniotic twins (MD twins) is 0.4% [4]. In contrast, the probability of conceiving twins after ovulation induction is 5%, and the probability of conceiving a twin after gonadotropin administration is 15%–20% [5]. In addition, the probability of conceiving a twin after ART is 3.2%, that of DD twins and MD twins after single embryo transfer is 0.63% and 1.4%, respectively [6,7], all of which are higher than the incidence of twins in spontaneous pregnancies.

Compared to singletons, twin pregnancies are more susceptible to perinatal complications, such as hypertensive disorder of pregnancy, and fetal growth restriction (FGR) [8,9], which are causes for preterm labor, defined as delivery before 37 weeks [8,10]. According to international demographics, 9.7% of singleton births are preterm, while 50% of multiple births are preterm [11]. Furthermore, twins have a higher perinatal mortality rate than singleton births [12,13]. In 1999, the infant mortality rates for singletons and twins in Japan were 2.9 and 15.5 per 1,000 live births, respectively, whereas in 2008, they were 2.2 and 8.6 per 1,000 live births, respectively [14]. In recent decades, perinatal care in Japan has improved, and the perinatal mortality rates for both singleton and twins have declined; however, the mortality rate for twins remains high. This high perinatal mortality rate is caused by the higher number of preterm births than singleton births [8,11]. Therefore, it is important to implement appropriate obstetric interventions promptly to reduce early neonatal and neonatal deaths in twins. This requires the accurate assessment of the growth and well-being of the fetus during pregnancy.

In general, the anthropometric reference values at birth by the length of pregnancy published in 2011 are used in most of the clinical settings in Japan to assess the child’s growth at birth [15]. This reference was developed for each sex and parity and was created using only the data on singleton births. However, the criteria are used for both singleton and multiple births.

Kato et al. developed a birth weight standard curve for 32,232 sets of twins (64,464 children) born in Japan between 1988 and 1991 [16]. The curve was based on vital statistics, but there has been no survey of all twins born in Japan since 1991. Therefore, we decided to analyze data on twins using data from the previous 22 years’ vital statistics. This study aims to create a standard birth weight reference curve specifically for twins and conduct a survey on neonatal and infant mortality.

## Materials and methods

We used birth and death certificates issued by the Ministry of Health, Labor, and Welfare (MHLW), which maintains the Japanese population’s statistics for children born in Japan between January 1, 1995, and December 31, 2016. The birth certificate included the mother’s nationality, date of birth, pregnancy history, age at delivery, birth weight, and other characteristics of the mother and child. In contrast, the death certificate included information, such as the date of birth and date of death. Since the birth certificates were not listed for twins, we needed to extract the twin pairs and check whether the pairs were correct. The date of birth of the two children, the delivery date of mothers, and the number of gestational weeks were used to match the twin pairs, and if these matched, the pair was considered a twin pair. Stillborn babies were excluded from this analysis. Data that lacked maternal or infant information, such as birth weight, gestational age, mother’s date of birth, or pregnancy history, were also excluded. In this study, triplets or other multiples were excluded.

**Fig 1:**
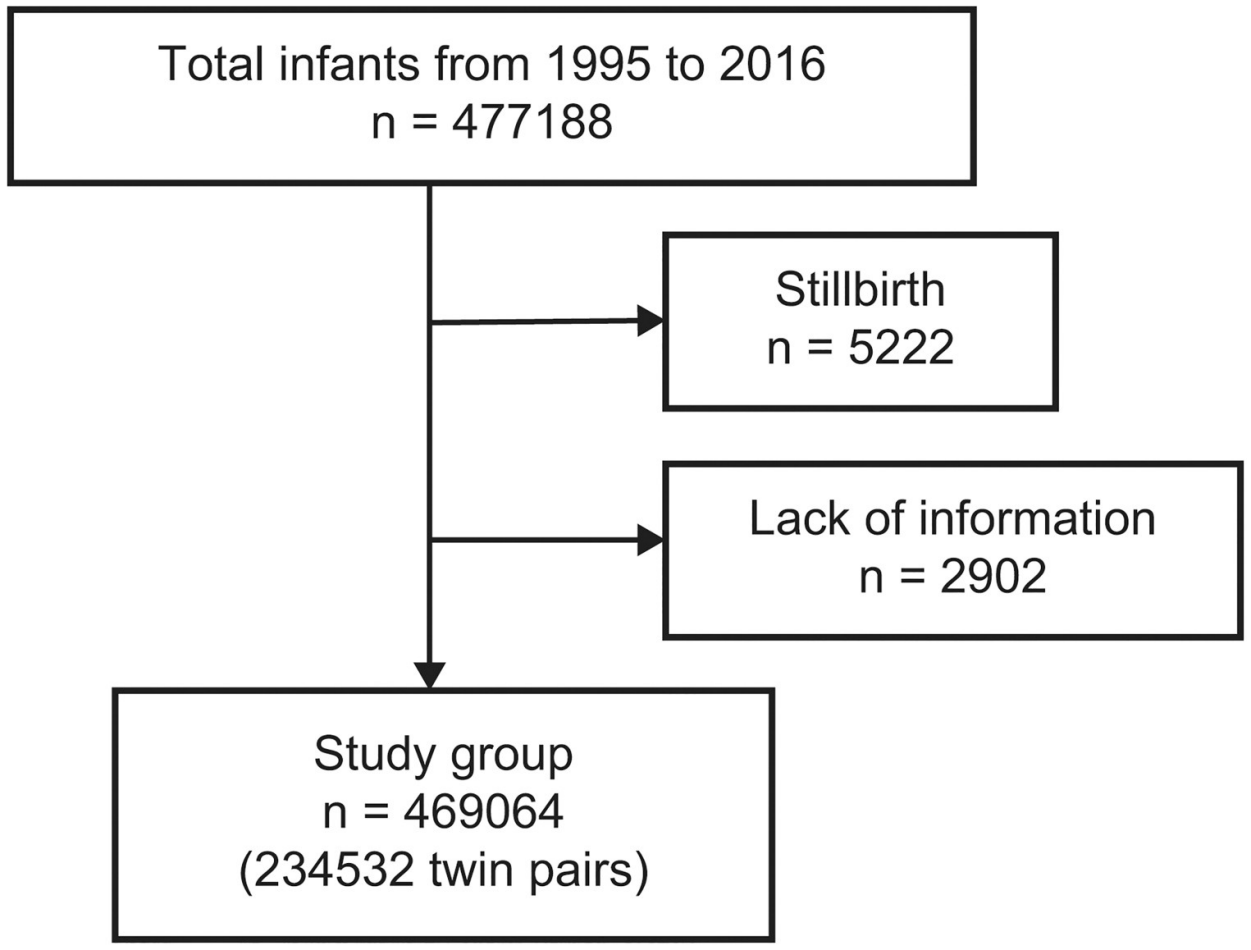
Flow chart for selecting twins.

The resulting matching rate of twin pairs was 96.6%. Neonates of foreign nationality were also excluded (Fig 1). Based on this information, the standard birth weight curves for each sex and parity were calculated using LMS methods. The 10^th^, 50^th^, and 90^th^ percentile birth weight values from 22 to 41 weeks were plotted.

For the mortality studies, the total number of early neonatal deaths, neonatal deaths, deaths between 29 days and one year of age, and infant deaths were calculated for each birth weight, week of delivery, and maternal age. Birth weight was classified as under 1000 g (defined as “extremely low birth weight” [ELBW]), 1000–1499 g, 1500–2499 g, 2500–3999 g, and over 4000 g. For maternal age, we also calculated the relationship between infant weight difference (discordance: <10%, 10 ≤ n <25%, ≤25%) and the number of weeks of delivery. Weight difference (discordance) was calculated using the following equation:

$$\text{Discordance}=\left[\left(\text{bigger}\;\text{infant}-\text{smaller}\;\text{infant}\right)/\text{bigger}\;\text{infant}\right]*100$$

### Statistical analysis

The incidence rate ratio (IRR), 95% confidence interval (95% CI), and p-values for neonatal and infant mortality were calculated to identify the risk factors for neonatal and infant mortality. The analysis included birth weight, week of delivery, and maternal age. IRR, 95% CI, and p-values were evaluated using the chi-squared test.

When drawing the birth weight standard curve, it is still crude. We used LMS Chart Maker light version 2.54 (Medical Research Council, London, UK) [17]. The LMS method describes the changes in distribution as L, M, S curves with the median (mu[M]), the coefficient of variation (σ[S]), and skewness (λ[L]). The likelihood equation was as follows:

$$\text{Z}=\left[\left(\text{X}/\text{M}\right)\text{L}-1\right]/\text{LS}=\text{M}*\;\left(1+\text{L}*\;\text{S}*\;\text{Z}\alpha \right)1/\text{L}$$

where Zα is the z-score corresponding to a given percentile (10^th^ percentile, 50^th^ percentile, and 90^th^ percentile), and X is the birth weight.

All statistical analyses were performed using R Core Team 2018 (R: R Foundation for Statistical Computing, Vienna, Austria). In this study, a p-value of <0.05 was considered statistically significant.

The research plan for this study was reviewed and approved by the Ethics Review Board of St. Luke’s International University (Document No. 2021–775). Informed consent was not required for the secondary use of the survey that was conducted using anonymized demographic birth and death certificates without identifying individuals. Moreover, all the data were anonymized. Thus, the Ethics Review Board of St. Luke’s International University decided to waive the requirement for a written informed consent.

## Results

Birth certificates from 1995–2016 were used for the analysis Table 1. Excluding stillbirths, 234,532 twin pairs (469,064 children) were included in the study. Among them, 51.1% of the twin pairs were born preterm. In terms of maternal age, 5.3% of mothers were under 25 years old, and 9.8% were over 40 years old. Regarding the birth weights, 67.2% were under 2,500 g (LBW), 6.51% were under 1,500 g (VLBW), and 2.2% were under 1,000 g (ELBW) (S1 Table).

**Table 1:** Main characteristics: Maternal and fetal data.

	n	%
**Total**		
Pairs	234,532	
Infants	469,064	
**Birth weight (g)**		
2500≤	153,688	32.8
Low birth weight	315,376	67.2
<1500 (Very low birth weight)	30,614	6.5
<1000 (Extremely low birth weight)	10,280	2.2
**Gestational week**		
Term	229,222	48.9
Preterm (less than 37w0d)	239,830	51.1
Late preterm (between 34w0d and 36w6d)	183,910	39.2
**Maternal age (years)**		
≤24	25,023	5.3
25–29	103,086	22
30–34	193,994	41.4
35–39	101,189	21.6
≥40	45,772	9.8

(24w6d: 24 weeks and 6 days; Preterm birth: Born between 22w0d and 36w6d; Late preterm birth: Born between 34w0d and 36w0d; Extremely low birth weight: Birth weight under 1000 g; Very low birth weight: Birth weight under 1500 g; Low birth weight: Birth weight under 2500 g).

**Fig 2:**
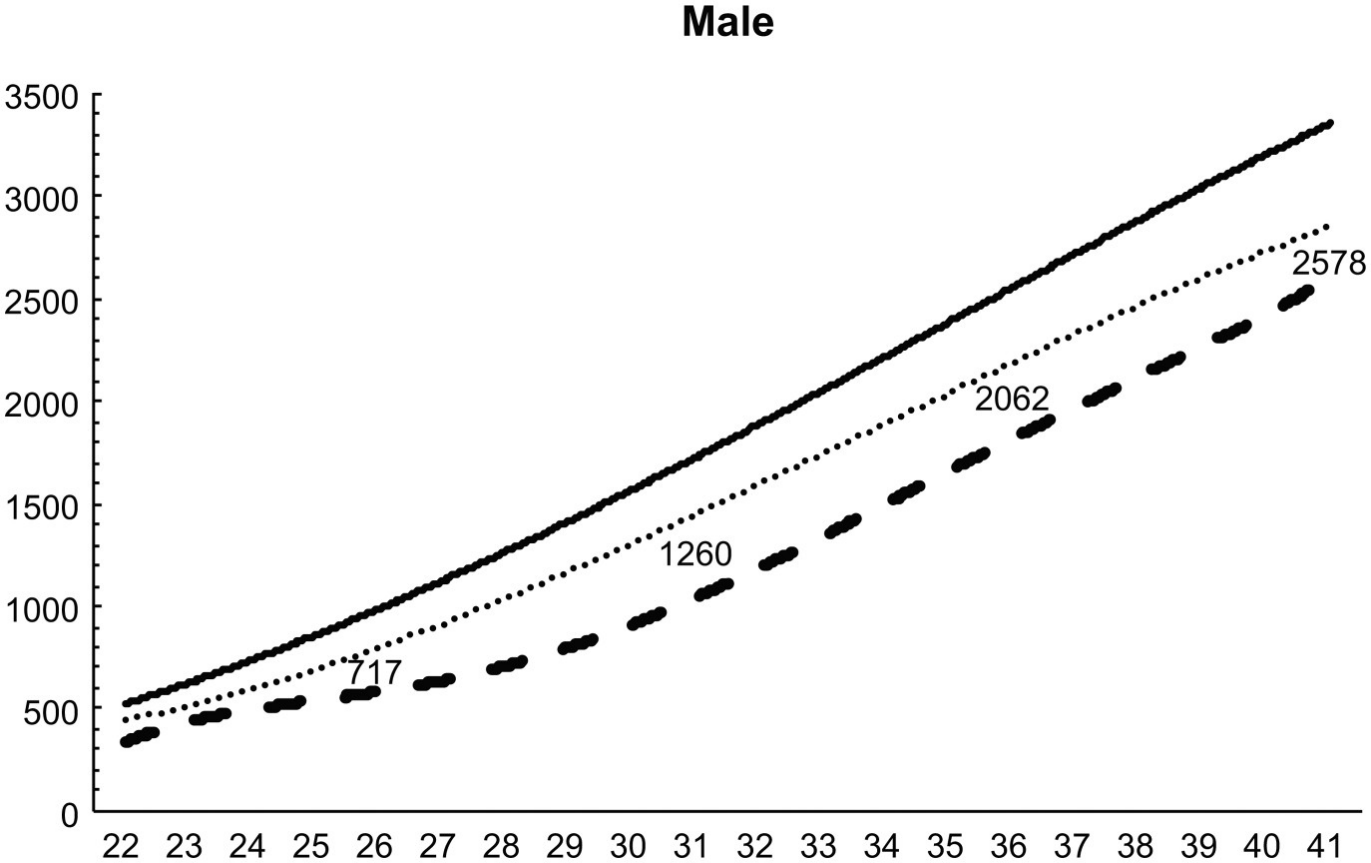
Birth weight reference curves for each week (male, primiparous).

**Fig 3:**
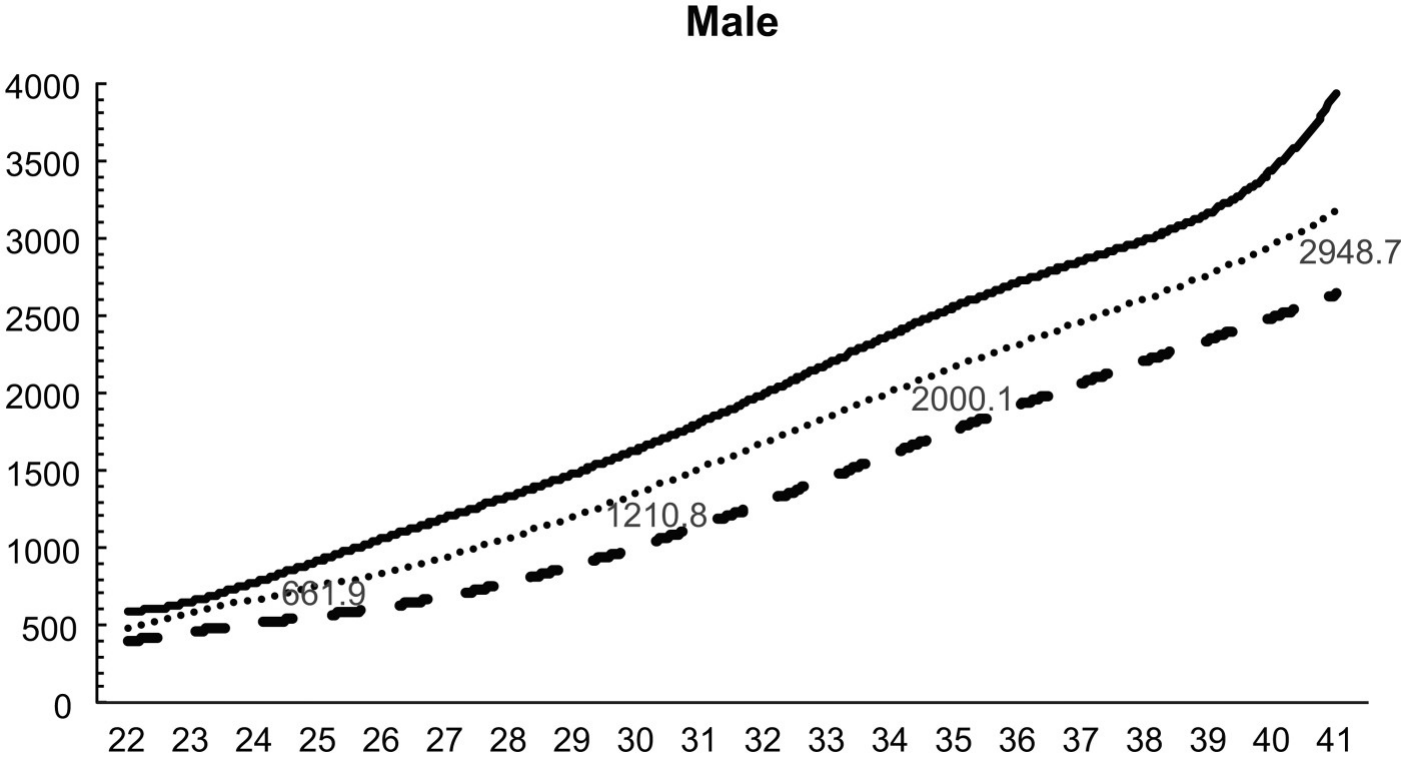
Birth weight reference curves for each week (male, multiparous).

**Fig 4:**
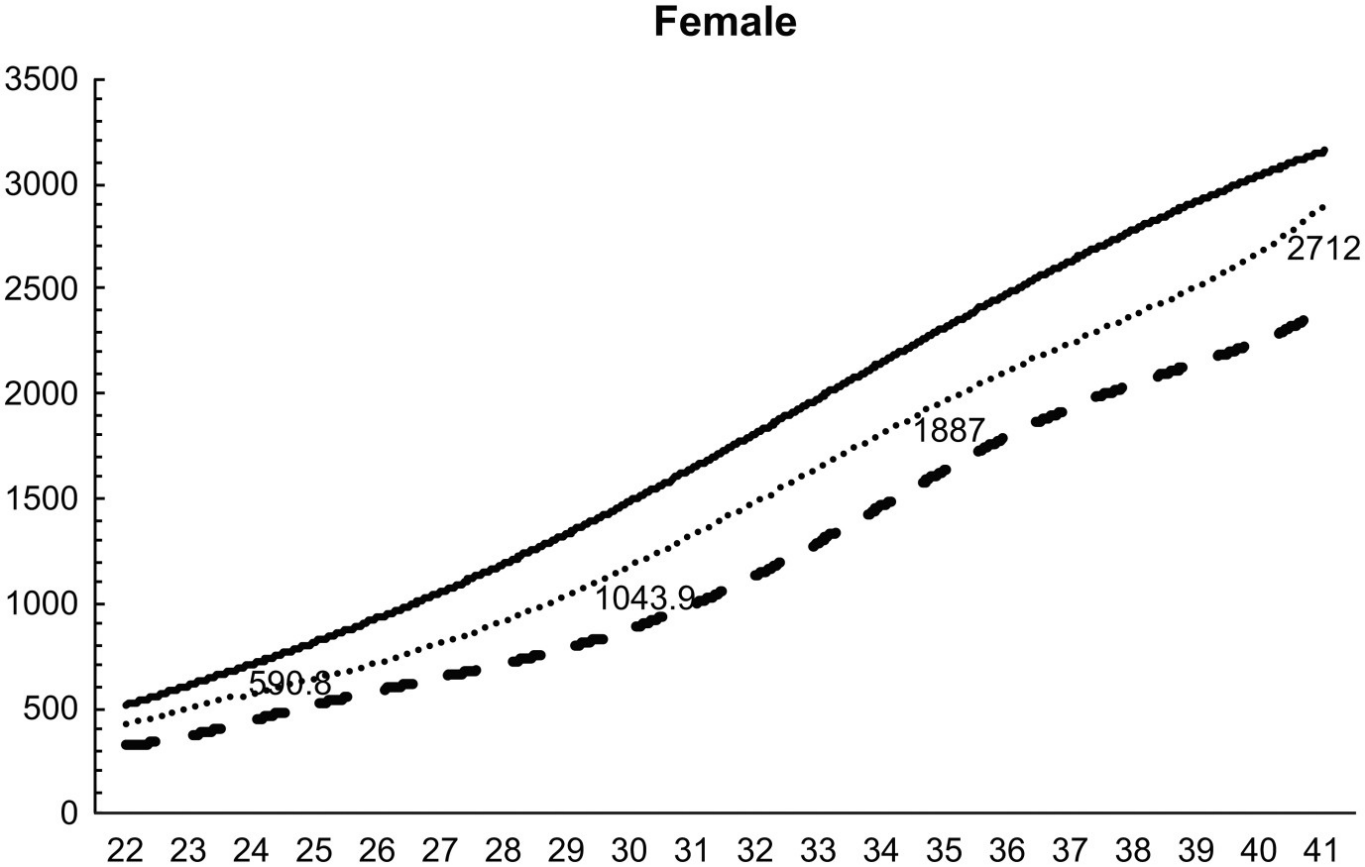
Birth weight reference curves for each week (female, primiparous).

**Fig 5:**
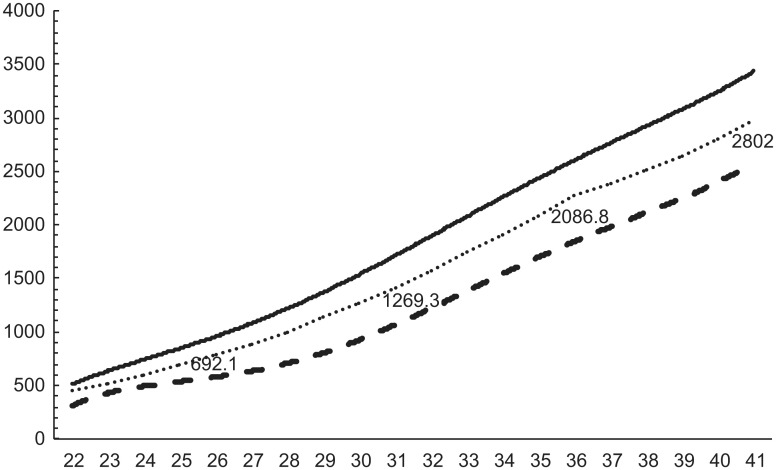
Birth weight reference curves for each week (female, multiparous).

Standardized birth weight reference curves for 22–41 weeks for both sexes and each parity were constructed using the LMS method (Figs 2–5). The 50^th^ percentile values were greater in boys than in girls at all weeks, for both first and second births. At 32 weeks, the difference in weight was over 200 g. Similarly, the difference in weight between males and females was 50–80 g until 30 weeks, but increased to more than 80 g at all weeks after 30 weeks.

Table 2 shows the relationship between birth weight and early neonatal death (END), late neonatal death (LND) within 8–28 days of birth, death between 29 days and 1 year of birth, and infant death (ID) (S2 Table). In the 1,500–2,499 g group, the mortality rate was the lowest (END: 0.2%, LND: 0.3%, ID: 0.6%). In the ≤1,499 g group, the mortality rate increased with smaller birth weight, and the proportion of infants dying within a month increased. The 2,500–3,999 g group had a lower mortality rate than the 1,500–2,499 g group. The 2,500–3,999 g group had an increased frequency of death between 29 days and one year of age (0.8%) compared to the 1,500–2499 g group. In the ≥4,000 g group, the END, LND, and ID were the highest among all the groups, and 92.3% of the children died within a year (END: 34.6%, LND: 38.4%, ID: 92.3%). The 2,500–3,999 g group had a higher frequency of death within 29 days to 1 year of birth than the 1,500–2,499 g group. The frequency of deaths in the 2,500–3,999 g group was higher than that in the 1,500–2,499 g group. We classified the 2,500–3,999g group by 500 g Table 3 (S2 Table). The results showed that the frequency of deaths in the 2,500–2,999 g group was similar to that in the 1,500–2,499 g group. In contrast, when the birth weight was over 3,000 g, the frequency of death was higher with each increase in birth weight.

**Table 2:** Relationship between early neonatal death/neonatal death and birth weight.

Birth weight (g)	≤999	1,000–1,499	1,500–2,499	2,500–3,999	4,000≤
**Total number of infants, n (%)**	10,280 (100)	20,334 (100)	284,762 (100)	153,622 (100)	26 (100)
**Number of deaths, n (%)**					
**<1 year of age (ID)**	1,191 (11.5)	494 (2.5)	1,571 (0.6)	1,861 (1.2)	24 (92.3)
**<7 days of age (END)**	610 (5.9)	256 (1.3)	606 (0.2)	473 (0.3)	9 (34.6)
**8–28 days of age (LND)**	842 (8.2)	320 (1.6)	770 (0.3)	641 (0.4)	10 (38.4)
**29 days to 1 year of age**	349 (3.3)	174 (0.9)	801 (0.3)	1,220 (0.8)	14 (53.9)

(END, early neonatal death; LND, late neonatal death; ID, infant death).

**Table 3:** Relationship between early neonatal death/neonatal death and birth weight (2500–3999 g).

Birth weight (g)	2,500–3,999	2,500–2,999	3,000–3,499	3,500–3,999
**Total number of infants, n (%)**	153,622 (100)	138,616 (100)	14,436 (100)	610 (100)
**Number of deaths, n (%)**				
**<1 year of age (ID)**	1,861 (1.2)	982 (0.7)	417 (2.9)	462 (75.7)
**<7 days of age (END)**	473 (0.3)	290 (0.2)	118 (1)	65 (9)
**8–28 days of age (LND)**	641 (0.4)	427 (0.3)	121 (0.8)	93 (15.2)
**29 days to 1 year of age**	1,220 (0.8)	555 (0.4)	296 (2.1)	369 (60.5)

(END, early neonatal death; LND, late neonatal death; ID, infant death).

Table 4 shows the relationship between the END, LND, deaths between 29 days and 1 year of age, and ID and the week of delivery (S2 Table). The lowest mortality rate was observed at 34 weeks, 0 days–36 weeks, 6 days (late preterm) (END: 0.2%, LND: 0.2%, ID: 0.4%). In the 22 week, 0 day–27 week, 6 day groups, 51.3% of the children who died within 1 year died within 7 days of birth and 72.3% died within 28 days of birth (END: 6.1%, LND: 8.6%, ID: 11.9%). The 37 week, 0 day–39 week, and 6 day groups had the same END (0.2%) and mildly increased LND (0.3%) compared with the 34 week, 0 days–36 week, 6 day groups, but the mortality rate within the period of 29 days–1 year of birth increased to 0.7% and the ID was 1%.

**Table 4:** Relationship between early neonatal death/neonatal death and gestational week.

	**22w0d–27w6d**	**28w0d–33w6d**	**34w0d–36w6d**	**37w0d–39w6d**	**40w0d–**
	9,090 (100)	46,830 (100)	183,910 (100)	222,410 (100)	6,812 (100)
	1,078 (11.9)	726 (1.5)	797 (0.4)	2,292 (1.0)	248 (3.6)
**<7 days of age (END)**	559 (6.1)	398 (0.8)	349 (0.2)	569 (0.2)	79 (1.1)
**8–28 days of age (LND)**	778 (8.6)	490 (1.0)	419 (0.2)	798 (0.3)	98 (1.4)
**29 days to 1year of age**	300 (3.3)	236 (0.5)	378 (0.2)	1494 (0.7)	150 (2.2)

(END, early neonatal death; LND, late neonatal death; ID, infant death).

Table 5 shows the relationship between END, LND, death within the period of 29 days–1 year after birth, and ID and maternal age. The 25–29 years old group had the lowest mortality rate (END: 0.3%, LND: 0.4%, ID: 0.9%). There was no clear difference in mortality among the other groups (END: 0.9%, LND: 0.8%, ID: 2.5%).

**Table 5:** Relationship between early neonatal death/neonatal death and maternal age.

Maternal age (years)	≤24	25–29	30–34	35–39	40≤
**Total number of infants, n (%)**	25,023 (100)	103,086 (100)	193,994 (100)	101,189 (100)	45,772 (100)
**Number of deaths, n (%)**					
**<1 year of age (ID)**	615 (2.5)	927 (0.9)	1,875 (1.0)	1,160 (1.1)	564 (1.2)
**<7 days of age (END)**	237 (0.9)	331 (0.3)	779 (0.4)	485 (0.5)	122 (0.5)
**8–28 days of age (LND)**	297 (0.8)	421 (0.4)	909 (0.5)	602 (0.6)	302 (0.7)
**29 days to 1 year of age**	318 (1.7)	506 (0.5)	966 (0.5)	558 (0.5)	262 (0.5)

(END, early neonatal death; ND, neonatal death; ID, infant death).

Table 6 shows the relationship between maternal age and weight difference (discordance) of the babies. In the maternal age under-25 group, 49.3% of mothers delivered twins with a 25% or more weight difference. In contrast, in the over-25 group, 20% of mothers delivered twins with a weight discordance of 25% or more.

**Table 6:** Relationship between maternal age and weight difference of babies who died within 1 year.

Discordance (%)	<10	10–24	25≤	Total
**Maternal age (years)**				
**≤24**	5,049 (20.2%)	7,644 (30.5%)	12,330 (49.3%)	25,023 (100%)
**25–29**	37,294 (36.2%)	42,332 (41.1%)	23,448 (22.7%)	103,074 (100%)
**30–34**	26,656 (13.7%)	123,878 (63.9%)	43,460 (22.4%)	193,994 (100%)
**35–39**	49,593 (49.0%)	30,600 (30.2%)	20,996 (20.7%)	101,189 (100%)
**40≤**	16,274 (35.6%)	20,418 (44.6%)	9,080 (19.8%)	45,772 (100%)
	134,866	224,872	109,314	469,052

Table 7 shows the relationship between maternal age and the number of weeks at which the baby was delivered. Mothers under 25 years old had the highest probability of delivery between 22 weeks, 0 days, and 27 weeks, 6 days (41.7%), and the lowest probability of delivery between 37 weeks, 0 days, and 39 weeks, 6 days (18.5%). In the over-25 group, 40%–60% of the women gave birth between 37 weeks, 0 days, and 39 weeks, 6 days.

**Table 7:** Relationship between maternal age and gestational week.

Gestational week	22w0d–27w6d	28w0d–33w6d	34w0d–36w6d	37w0d–39w6d	40w0d–	Total
**Maternal age (years)**						
**≤24 (%)**	3,788 (15.1)	4,086 (16.3)	12,401 (49.6)	4,640 (18.5)	108 (0.4)	25,023 (100)
**25–29 (%)**	3,126 (3)	13,320 (12.9)	42,832 (41.5)	42,558 (41.3)	1,238 (1.2)	103,074 (100)
**30–34 (%)**	1,532 (0.8)	26,472 (13.6)	48,244 (24.9)	113,882 (58.7)	3,864 (2)	193,994 (100)
**35–39 (%)**	256 (0.3)	2,532 (2.5)	56,221 (55.6)	40,886 (40.4)	1,294 (1.3)	101,189 (100)
**40≤ (%)**	388 (0.8)	420 (0.9)	24,212 (52.9)	20,444 (44.7)	308 (0.7)	45,772 (100)

Table 8 shows the IRR, 95% CI, and p-value for ND and ID. In terms of birth weight, the VLBW group had a higher risk of ND and ID, and the group that delivered babies that weighed 2,500 g or more had a higher risk of ID (ID: IRR = 1.1, 95% CI = 1.3–1.85, p < 0.001).

**Table 8:** Adjusted risks of ND and ID.

	ND				ID			
	IRR	95% CI		p-value	IRR	95% CI		p-value
**Birth weight (g)**								
**≤999**	2.90	1.98	2.85	<0.0001	3.20	1.92	2.77	<0.0001
**1,000–1,499**	2.34	2.10	2.40	<0.0001	2.51	1.67	2.97	<0.0001
**1,500–2,499**	1	reference			1	reference		
**2,500–3,999**	0.96	0.95	1.13	0.2	1.1	1.33	1.85	0.002
**4,000≤**	2.39	1.69	2.41	<0.0001	3.14	1.58	1.89	<0.0001
**Gestational week**								
**22w0d–27w6d**	2.94	3.4	4.2	<0.0001	3.37	1.92	2.4	<0.0001
**28w0d–33w6d**	2.3	1.8	2.77	<0.0001	2.8	1.63	2.89	<0.0001
**34w0d–36w6d**	1	reference			1	reference		
**37w0d–39w6d**	0.72	0.51	1.23	0.04	1.3	1.21	1.93	0.04
**40w0d–**	2.33	1	1.6	<0.0001	2.18	1.9	2.37	<0.0001
**Maternal age (years)**								
**≤24**	1.815	1.688	1.964	0.02	1.82	1.71	1.95	0.01
**25–29**	1	reference			1	reference		
**30–34**	1.088	0.966	1.226	0.17	1.12	1.01	1.25	0.07
**35–39**	1.161	1.021	1.321	0.06	1.17	1.05	1.31	0.06
**40≤**	1.176	0.971	1.424	0.10	1.17	0.99	1.38	0.07

(ND, neonatal death; ID, infant death).

Comparison control of each factor is shown.

Birth weight: 1500–2499 g.

Gestational week: Late preterm.

Maternal age: 25–29 years.

Regarding the relationship between mortality and the number of weeks of delivery, the IRR was significantly higher for both ND and ID when delivery occurred between 22 weeks, 0 days and 33 weeks, 6 days. The IRR was significantly higher in ID when delivery occurred after 37 weeks 0 days (ID: IRR = 1.3, 95% CI = 1.21–1.93, p-value = 0.04). Regarding maternal age, the under-25 group had significantly higher IRR in both END and ND (END: IRR = 1.82, 95% CI = 1.68–1.96, p-value = 0.02; ND: IRR = 1.82 95% CI = 1.71–1.95, p-value = 0.01).

## Discussion

In this study, we investigated the increasing trend of twin births using data from the MHLW. We constructed standardized birth weight curves for male and female babies by menarche and first childbirth and identified the risk of ND and ID. The results showed that the birth weight of male babies was higher than that of female babies at all weeks, regardless of parity. The lowest frequency of ND and ID was observed in twins born at a birth weight of 1,500–2,499 g, late preterm deliveries, and maternal age at delivery of 25–29 years.

The anthropometric reference values at birth by the length of pregnancy currently used in Japan were developed by including only singleton births [15]. We compared the 50^th^ percentile values of that graph with the 50^th^ percentile values of our birth weight reference curve for twins born between 22 and 41 weeks. Our 50^th^ percentile values were smaller at all weeks, regardless of sex or pregnancy history. The birth weight of twins is smaller than that of singletons [18], and our study showed the same result.

We then compared the 50^th^ percentile values of our birth weight reference curve with those of a birth weight reference curve based on a survey of all births between 1988 and 1991 [16]. Our graph showed that birth weights were 30–50 g lower regardless of sex or pregnancy history, except for babies born at 40 and 41 weeks. Birth weight has decreased year by year in singleton births in Japan [19], which is related to the changes in the maternal background and the transition to ART. The decrease in birth weight in twins was discussed in the same way as in singleton births. The number of Japanese women aged 20–39 years with a body mass index (BMI) <18.5 has increased in the last 30 years [20]. Furthermore, many pregnant women have not gained enough weight during pregnancy [21]. Low BMI before pregnancy and poor weight gain during pregnancy has been associated with preterm birth and low birth weight [21,22]. In recent years, there has been an increase in the number of children born via ART in Japan [23]. Twins born via ART have significantly higher preterm birth rates, very low birth weight, neonatal mortality, artificial respiration, and respiratory distress syndrome compared to twins conceived spontaneously [24,25]. Therefore, the increased number of ART-induced twins born, recently compared to those born in 1988–1991, can be cited as one of the reasons for the decrease in the birth weight of twins.

The higher frequency of deaths after 29 days of age in the 2,500–3,999 g birth weight group (0.3%) compared to the 1,500–2,499 g group (0.3%) is due to the large proportion of deaths in children with birth weights of 3,000 g and above, especially over 3,500 g. Children born large for gestational age have a higher mortality rate than those born appropriate for gestational age. Although we could not classify by sex, there is a high probability that MD twins are among those born at 3,500 g or more.

In twins, late preterm delivery had the lowest frequency of ID. A comparison of neonatal outcomes between singleton and twin babies born in the late preterm showed that the duration of hospitalization was significantly longer in twins than in singletons. However, the neonatal epidemic and mortality rates remained the same [26]. Twins may receive appropriate neonatal care during their long hospital stay, which improves their prognoses. Mothers of twins receive better guidance after discharge, such as appropriate guidance on sleeping on the back to prevent sudden ID syndrome (SIDS). It is also possible that many children born after 37 weeks and 0 days may be considered healthy after birth and may be discharged from the hospital without the mothers receiving an adequate education.

Mothers younger than 25 years had the highest frequency of ND and ID. In general, mothers under 20 years of age are 1.78 times more likely to experience preterm labor than mothers aged 20–34 [27]. In the present study, mothers under 25 years of age were the most likely to have a preterm birth. Furthermore, infants born to mothers younger than 20 years had significantly more SGA [28] (cOR: 1.92, 95% CI: 1.17–3.17). Mothers over 40 years of age are more likely to become pregnant via ART [29].

A report published in 2018 stated that younger women were more likely to become pregnant with predominantly MD twins than older women [30,31]. In our present study, we also found that mothers younger than 25 years had a higher rate of delivering twins with high weight differences and were also more prone to preterm delivery. Although we could not classify the nature of the deaths, we believe that mothers under 25 years of age have a higher frequency of ND and ID for these reasons.

Our study is reliable because we used data from all births and deaths recorded in the MHLW birth and death certificates for the past 22 years. Few studies have analyzed all births for such a long time. The birth weight of twins had decreased compared to 1988. We also found that neonatal and infant deaths were lower in deliveries with birth weights between 1,500–2,499 g and late preterm deliveries. The mothers of these infants likely received appropriate guidance during hospitalization, including guidance regarding sleeping on the back. The mothers of infants born at a birth weight of more than 2,500 g or born after 37 weeks should also receive appropriate guidance on SIDS prevention during hospitalization.

This study had some limitations. Information not included in the birth and death certificates is missing from this study. For example, the chorionic and amniotic nature of the twins was not classified. As mentioned above, the risk of perinatal death differs greatly between DD and MD twins, and it is necessary to analyze them separately for more accurate risk analyses. Using the MHLW data, it was not possible to determine whether infants had anomalies. In addition, information regarding the maternal socioeconomic background and the use of fertility treatment was not available. In particular, the maternal background may affect the analysis of age risk. In recent years, a perinatal database has accumulated information on the maternal background, mainly in higher medical institutions. Because of the characteristics of twins, they are often managed by higher-order medical institutions, and therefore, more detailed information on twins can be covered by perinatal databases. In the future, we would like to use the perinatal database to examine the risk of infant mortality in twins, taking into account various other confounding factors into account.

## Conclusions

Standard birth weight curves for twins were generated. The birth weight of twins was found to be decreasing in both sexes, reflecting the social background of the mother and the transition to infertility treatment. At the same time, IDs were calculated by birth weight, week of delivery, and age, respectively. Twins have the best prognosis when delivered at a birth weight of 1,500–2,499 g and when born at late preterm. Home management guidance for twin full-term and normal birth weight infants may reduce infant mortality.

## Supporting information

S1 Table Total number of births for each year from 1995 to 2016. (XLSX)

S2 Table Early neonatal, neonatal, and infant mortality based on birth weight and gestational week. (XLSX)
